# Evaluation of constitutive iron reductase (*AtFRO2*) expression on mineral accumulation and distribution in soybean (*Glycine max.* L)

**DOI:** 10.3389/fpls.2014.00112

**Published:** 2014-04-03

**Authors:** Marta W. Vasconcelos, Thomas E. Clemente, Michael A. Grusak

**Affiliations:** ^1^Centro de Biotecnologia e Química Fina, Escola Superior de Biotecnologia, Centro Regional do Porto da Universidade Católica PortuguesaPorto, Portugal; ^2^Department of Pediatrics, USDA-ARS Children’s Nutrition Research Center, Baylor College of MedicineHouston, TX, USA; ^3^Center for Biotechnology – Plant Science Initiative, University of Nebraska-LincolnLincoln, NE, USA

**Keywords:** FRO2, iron, mineral nutrition, soybean, transgenic

## Abstract

Iron is an important micronutrient in human and plant nutrition. Adequate iron nutrition during crop production is central for assuring appropriate iron concentrations in the harvestable organs, for human food or animal feed. The whole-plant movement of iron involves several processes, including the reduction of ferric to ferrous iron at several locations throughout the plant, prior to transmembrane trafficking of ferrous iron. In this study, soybean plants that constitutively expressed the AtFRO2 iron reductase gene were analyzed for leaf iron reductase activity, as well as the effect of this transgene’s expression on root, leaf, pod wall, and seed mineral concentrations. High Fe supply, in combination with the constitutive expression of AtFRO2, resulted in significantly higher concentrations of different minerals in roots (K, P, Zn, Ca, Ni, Mg, and Mo), pod walls (Fe, K, P, Cu, and Ni), leaves (Fe, P, Cu, Ca, Ni, and Mg) and seeds (Fe, Zn, Cu, and Ni). Leaf and pod wall iron concentrations increased as much as 500% in transgenic plants, while seed iron concentrations only increased by 10%, suggesting that factors other than leaf and pod wall reductase activity were limiting the translocation of iron to seeds. Protoplasts isolated from transgenic leaves had three-fold higher reductase activity than controls. Expression levels of the iron storage protein, ferritin, were higher in the transgenic leaves than in wild-type, suggesting that the excess iron may be stored as ferritin in the leaves and therefore unavailable for phloem loading and delivery to the seeds. Also, citrate and malate levels in the roots and leaves of transgenic plants were significantly higher than in wild-type, suggesting that organic acid production could be related to the increased accumulation of minerals in roots, leaves, and pod walls, but not in the seeds. All together, these results suggest a more ubiquitous role for the iron reductase in whole-plant mineral accumulation and distribution.

## INTRODUCTION

Iron is one of the most important micronutrients in human and plant nutrition, and a suitable level of iron nutrition in plants is vital to providing adequate concentrations of this mineral in the harvestable plant organs for human food or animal feed. Researchers have been interested in creating plant foods that are nutrient-dense (to guarantee “nutrient security”) in iron and other minerals (Carvalho and Vasconcelos, 2013), using an approach referred to as biofortification. Soybean, being an important plant food in several parts of the world, would be a suitable target for biofortification programs. However, in soybean, most biofortification strategies have aimed at increasing sulfur amino acids (Dinkins et al., 2001) and vitamins, such as α-tocopherol (Dwiyanti et al., 2011), and not at increasing mineral concentrations.

To create crop plants with more minerals, researchers must boost their mobilization and uptake from the soil, improve their movement to the edible portions of the plant and ultimately, enhance their storage in those tissues. For this, it is essential to understand the relevant contributions of the mineral transport system throughout the plant as well as the regulatory system that controls it.

One possible strategy is to utilize a “bottom up” approach, where scientists enhance the mechanism of iron uptake from the roots, and “hope” that the additional iron will be mobilized and stored in the edible parts of the plant. Mineral elements traverse the root via apoplastic and/or symplastic pathways to the stele, where they are loaded into the xylem for transport to the shoot (White and Broadley, 2009). In the plant kingdom there are two main strategies for iron uptake. Dicots, such as soybean, and non-graminaceous monocots, respond to iron limiting environments by induction of the Strategy I mechanism (Römheld and Marschner, 1986; Marschner, 1995), which involves several processes at the root membrane such as the expression of active proton pumps (AHA2) to increase solubility of ferric (Fe^+3^) iron, a ferric reductase (FRO2) to generate ferrous (Fe^+2^) iron, and an iron transporter (IRT1) to take up this reduced, available iron from the rhizosphere (Hell and Stephan, 2003). This uptake system depends on the activity of specific transcription factors, such as FIT and bHLH proteins. Recent work in *Arabidopsis* and *Medicago* has shown that Strategy I plants also produce species-specific iron deficiency-elicited compounds, namely phenolics and flavins (Rodríguez-Celma et al., 2013) such as scopoletins (Fourcroy et al., 2013). Monocots utilize the Strategy II mechanism, in which there is extrusion of phytosiderophores that chelate iron and then are taken up as an iron-chelate complex by root specific transporters. Several comprehensive reviews are currently available on this topic (Curie and Briat, 2003; Walker and Waters, 2011; Hindt and Guerinot, 2012; Ivanov et al., 2012; Kobayashi and Nishizawa, 2012).

The *Arabidopsis* FRO2 gene is a member of the FRO gene family and encodes an iron-deficiency inducible iron reductase responsible for reducing iron at the root surface (Yi and Guerinot, 1996; Robinson et al., 1999; Connolly et al., 2003). Once inside the roots, iron is transported via the xylem to the vegetative tissues in response to transpirational activity from various organs. It is thought that iron is transported in the xylem as a complex with organic acids (OA) such as citrate (Tiffin, 1966; Rellán-Álvarez et al., 2010), and that nicotianamine (NA) also may play an important role in this process (Curie et al., 2009; Ivanov et al., 2012).

The role of iron reduction in the roots is well documented (Robinson et al., 1999; Connolly et al., 2003; Mukherjee et al., 2006); however, the role of the reductase in the leaf, fruit, and grain is still unclear. It is thought that iron reduction is necessary to reduce ferric iron in the aerial parts of the plant before being transported into the leaf cells (Larbi et al., 2001; Feng et al., 2006). It was found that AtFRO7 localizes to the chloroplast and is required for efficient photosynthesis in young seedlings and for survival under iron-limiting conditions (Jeong et al., 2008). Iron reductase activity has been detected in leaves of different plant species such as sunflower (de la Guardia and Alcántara, 1996), *Vigna unguiculata* (Brüggemann et al., 1993) and sugar beet (Gonzalez-Vallejo et al., 2000; Larbi et al., 2001).

Once in the leaves, iron is used in diverse biochemical processes, or it can be stored for future use. The storage form of iron and the organelles where it is accumulated is only partially defined. It is thought that most of the iron will be stored as ferritin in the plastids (chloroplasts contain up to 90% of the leaf cell iron), with about half in the stroma and half in the thylakoid membranes. The remaining iron pool will probably be found in the vacuole (Thomine et al., 2003). There also seems to be a role for the reductase in the transport to and eventual accumulation of iron in seeds (Grusak, 1995). A pea mutant (*dgl*) was identified that has uncontrolled hyperaccumulation of Fe in vegetative tissues, and that has the ability to accumulate up to 3-fold higher concentrations of Fe in the seeds (Marentes and Grusak, 1998). Another pea mutant, *brz* (bronze leaves) also has been shown to have hyperaccumulation of iron in the leaves, but it does not move excess iron into the seeds (Grusak, 1995). Unfortunately, there are no data on the possible role of leaf iron reduction in the transport of iron to the developing seeds.

There is also very limited information on the common regulatory mechanisms that co-regulate the uptake, transport and accumulation of iron and other minerals in the plant. Several common transporters have been identified, but only limited evidence is available on the effects of higher Fe intake on the dynamics of other minerals, such as zinc (Zn), copper (Cu), manganese (Mn), magnesium (Mg), phosphorous (P), potassium (K), nickel (Ni), molybdenum (Mo), or calcium (Ca). All of these also play important roles in human and animal nutrition, and enhanced accumulation of more than one mineral could be a cost effective strategy in biofortification programs.

Ectopic expression of the *Arabidopsis thaliana* ferric chelate reductase, *AtFRO2* (Robinson et al., 1999) in transgenic soybean conferred increased iron reduction in the roots and enhanced tolerance towards iron deficiency chlorosis (Vasconcelos et al., 2006). To determine if the reductase activity is a rate-limiting step for seed mineral acquisition, and to establish *AtFRO2* as a possible molecular tool to be used in biofortification programs, we functionally analyzed the transgenic soybean line 392-3 carrying the *Arabidopsis FRO2* gene under the 35S constitutive promoter for leaf iron reductase activity and general whole-plant mineral distribution.

## MATERIALS AND METHODS

### PLANT MATERIAL

Soybean (*Glycine max* L.) genotypes “Thorne” wild-type (WT) and transgenic homozygous line 392-3 carrying the *FRO2* gene from *Arabidopsis thaliana* under the constitutive 35S promoter (Vasconcelos et al., 2006) were used in this study. Plants were grown in a controlled environment chamber with 16-h, 20°C-day and 8-h, 15°C-night. Relative humidity was maintained at 50% and photon flux density during the day was 350 μmol m^-2^ s^-1^, supplied by a mixture of incandescent bulbs and fluorescent lamps. Seeds of control and homozygous transgenic plants in T_3_ generation were first germinated in beakers with wet filter paper for 4 days before being transferred to hydroponics solution (four plants per 4.5 l) with different Fe treatments. The standard solution for hydroponically grown plants contained: 0.8 mM Ca(NO_3_)_2_, 1.2 mM KNO_3_, 0.2 mM MgSO_4_, 0.3 mM NH_4_H_2_PO_4_, 25 μM CaCl_2_, 25 μM H_3_BO_3_, 0.5 μM MnSO_4_, 2.0 μM ZnSO_4_, 0.5 μM CuSO_4_, 0.5 μM H_2_MoO_4_, and 0.1 μM NiSO_4_. For various studies, plants were grown either at 0, 10, 32, or 100 μM Fe(III)-EDDHA [ethylenediamine-N,N’bis(o-hydroxyphenyl)acetic acid]. All nutrients were buffered with 1 mM MES (2,4-morpholino-ethane sulfonic acid), pH 5.5 and growth solutions were changed weekly until full maturity (FM) of the plants.

For mineral analysis, tissues were collected at three developmental stages: 40 (Grain-Fill I), 80 (Grain-Fill II), and 120 (Grain-Fill III) days after the initiation of flowering.

### LEAF PROTOPLAST ISOLATION AND REDUCTION ACTIVITY

Trifoliate leaves of Thorne and 392-3 were collected at two weeks of age from plants grown with 0, 10, 32, or 100 μM Fe(III)-EDDHA. Protoplasts were isolated using a method modified from Larbi et al. (2001). Specifically, leaf tissue of a known surface area was cut and placed in Solution A (500 mM D-sorbitol, 1 mM CaCl_2_, 2% [w/v] cellulase, 0.3% macerozyme, 0.1% pectolyase, 5 mM MES, pH 5.5). The macerate was incubated at 27°C for 3 h on an orbital shaker at 70 rpm, in the dark. Protoplasts were filtered, centrifuged, washed with Solution B (500 mM D-sorbitol, 1 mM CaCl_2_ and 5 mM MES, pH 6.0), isolated after sucrose layering and centrifugation, sized, and counted for viability. Protoplasts were sized by using a calibrated eyepiece fitted to a microscope (Nikon, Japan, 40× objective). The number of protoplasts per unit volume was determined with a hemacytometer (Hausser Scientific, Horsham, PA, USA), and viability of the protoplasts was calculated by incubation of the protoplast suspension in 0.04% (w/v) Evans Blue for 5 min, and determining the number of protoplasts excluding the dye. A 500 μl volume of leaf protoplast solution was added to 500 μl of Solution B (described above) supplemented with 800 μM BPDS and 800 μM Fe(III)-EDTA. Tubes were agitated for 45 min at 27°C, centrifuged (2 min at 12,000 *g*) and absorbances read at 535 nm. The extinction coefficient of Fe(II)-BPDS_3_ (22.14 mM^-1^ cm^-1^) was used to calculate the Fe reduction rates; values are presented on protoplast surface area basis (nmol of Fe reduced μm^-2^ s^-1^).

### TISSUE ELEMENTAL ANALYSIS

All tissues were harvested and dried overnight in a 60°C oven. Samples were then digested overnight in borosilicate glass tubes by adding 4 ml of redistilled 98.8% HNO_3_ and 1 ml of concentrated trace metal grade HClO_4_. Samples were heated at 100°C for 1 h, 150°C for 1 h, 180°C for 1½ h and then at 210°C to dryness. Digestions were performed using a heating block (Model 1016, Tecator, Höganäs, Sweden) with an exhaust-collecting manifold. Digests were resuspended in 10 ml of redistilled 2% HNO_3_. Elemental analysis was performed using inductively coupled plasma – optical emission spectroscopy (ICP-OES; CIROS ICP Model FCE12; Spectro, Kleve, Germany). Five plants were grown for each treatment as described before. Material from each plant was ground and five independent digestions were performed prior to ICP-OES analysis.

### *AtFRO2* AND *GmFer* EXPRESSION ANALYSIS

RNA from roots, leaves, seeds, root tips, pod walls, and petals of control and transgenic plants grown hydroponically under various iron concentrations (see above) was isolated using the RNA-easy kit (Qiagen Inc., Valencia, CA, USA) according to the manufacturer’s instructions. RNA was isolated from 3 plants in each treatment. The experiments were repeated twice, and PCR reactions were performed at least in triplicate. Possible contaminating genomic DNA was removed with the TURBO DNA-*free*^TM^ kit from Ambion (Ambion Inc., Austin, TX, USA) following the manufacturer’s instructions. Total RNA (0.5 μg) were subjected to reverse transcription (RT) with an anchored oligo (dT) primer and 200 units superscript II reverse transcriptase (Invitrogen, Carlsbad, CA, USA) in a volume of 20 μl according to the manufacturer’s instructions. PCR reactions were carried out with 1.5 μl of the RT reaction solutions. Additional reaction components were: 10 mM polymerase buffer, 1 mM dNTP’s, 0.1 units Taq polymerase (Clontech, Palo Alto, CA, USA) and 1 μM specific primers. All primers surrounded an intron so that genomic DNA was clearly distinguished from cDNA-derived products. The following primer sets were designed: *ferritin* (*GmFer*), 5′-ACTTGCTCTGTTTCTCTGAGC-3′ (forward), 5′–CGCTAGACGGTGTGACACGT-3′ (reverse); *ubiquitin* (*GmUbq*), 5′-GGGTTTTAAGCTCGTTGT-3′ (forward) and 5′-GGACACATTGAGTTCAAC-3′ (reverse). The number of cycles in each PCR reaction was 28, with 58°C annealing temperature. Amplified products from 10 μl of PCR reaction were visualized on a 1% TAE agarose gel containing ethidium bromide. Bands were photographed using the Quantity One 4.5.1 Chemidoc EQ^TM^ Software System (Biorad, CA, USA).

Quantitative RT-PCR (Q-RT PCR) was performed for *AtFRO2* transcript quantification in different plant tissues. Reactions were carried out in an ABI PRISM® 7700 sequence detector using TaqMan One Step PCR master Mix reagents Kit and an ABI PRISM® 96-well optical reaction plate (all from Applied Biosystems, Foster City, CA, USA). Validation of the Q-RT PCR methodology was performed in order to find the appropriate RNA concentration at which there is a linear correlation between ribosomal RNA control (*18S rRNA)* and *AtFRO2* transcription. Reactions were carried out with 0.2 ng/μl RNA to a final volume of reaction of 25 μl. Specific primers designed for *AtFRO2* were: 5′-CGTATCAAGTTTGGAACATCCACTT-3′ (forward) and 5′-CCATCATTGGGAACATATACATGAA-3′ (reverse), amplifying the TaqMan Probe *AtFRO2* sequence of 5′-AAGTTTGGAACATCCACTTATTTTGGTGCCA-3′. For signal detection and quantification, Applied Biosystems-Sequence Detection Systems 1.9.1 was used (Applied Biosystems, Foster City, CA, USA). The following standard thermal profile was used for all PCRs: 40 cycles starting at 48°C 30 min, 95°C 10 min, 95°C 15 s and 60°C for 1 min. The δCt ± SD and mean transcript level were calculated between four technical replicates from two experiments. To generate a baseline-subtracted plot of the logarithmic increase in fluorescence signal (δR*_n_*) versus cycle number, baseline data were collected between cycles 3 and 15. All amplifications were analyzed with an R*_n_* threshold of 0.02 to obtain the C_T_ (threshold cycle) values.

### ORGANIC ACID ANALYSIS

Leaf and root tissue of control (Thorne) and transgenic (392-3) soybean plants grown hydroponically at 0, 10, 32, or 100 μM Fe (III)-EDDHA for 2 weeks were used for citrate and malate analysis. Five samples of each tissue were frozen in liquid nitrogen and ground in a ceramic mortar and pestle with 8 mM sulfuric acid. Homogenates were boiled for 30 min, filtered with a 0.2 μm filter (Falcon, USA), taken to a final volume of 2 ml with 8 mM sulfuric acid and kept at -80°C until HPLC analysis.

Organic anions were analyzed with an Acclaim OA 5 μm ion-exchange column (250 × 4 mm, DIONEX, TX, USA) with an HPLC system (ICS 3000 Ion Chromatography System, DIONEX, Houston, TX, USA), and Chromeleon software. Samples were manually injected (10-μl loop). Mobile phase (100 mM Na_2_SO_4_, pH 2.65) was pumped with an isocratic 0.5 ml min^-1^ flow rate. Organic anions were detected at 210 nm. Peaks corresponding to citrate and malate were identified by comparison of their retention times with those of known standards from Bio-Rad and Sigma (St. Louis). Quantification was made with known amounts of each organic anion using peak areas.

### STATISTICAL ANALYSIS

Welch’s *t*-test and the Dunnett-*C* test to compare the means were used to compare the leaf, pod wall, root, stem and seed nutrient levels between transgenic and WT plants. A Pearson’s correlation analysis with four significance levels (*P* < 0.05; *P* < 0.01; *P* < 0.001, and *P* < 0.0001) was performed to determine the correlation between 10 different minerals in different tissue types. The mineral data used for the correlation study integrated the ICP results from WT and 392-3 plants grown to maturity at the three iron concentrations, and at the three harvest dates. All statistical analyses were performed using GraphPad Prism 6, version 6.1 (La Jolla, CA, USA).

## RESULTS

### PLANT ORGAN WEIGHT

**FIGURE 1:**
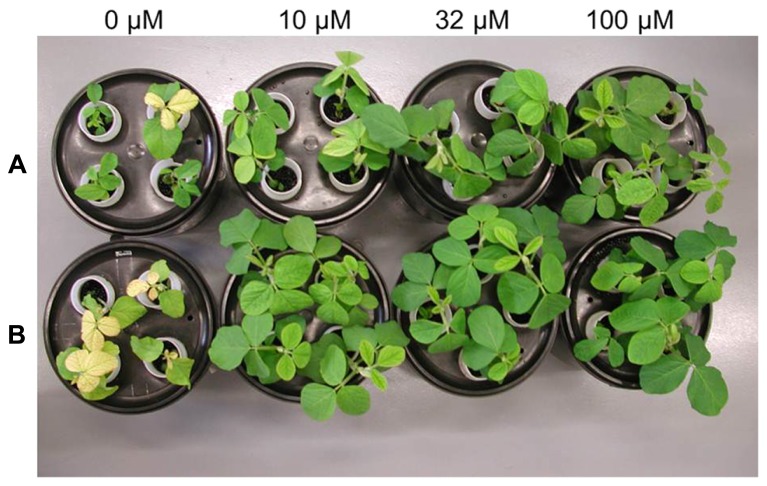
**Wild-type (Panel A) and transgenic 392-3 (Panel B) soybean plants at 14 days of hydroponic growth supplemented with 0, 10, 32, or 100 μM Fe(III) EDDHA (left to right)**.

Fourteen days after transfer to the hydroponics solution, WT and 392-3 plants appeared similar to each other, in terms of plant size and leaf color, particularly at 100 μM Fe(III)-EDDHA (**Figure 1**). However, both WT and 392-3 plants grown in the complete absence of iron were already showing severe signs of chlorosis (**Figure 1**); therefore, this treatment had to be discontinued for the analysis that involved growing the plants to FM.

**FIGURE 2:**
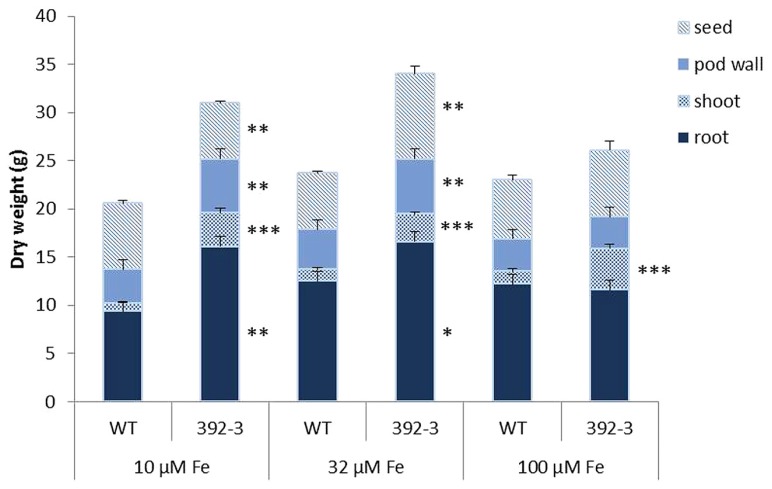
**Dry weight of seeds, pod walls, shoot (leaf and stem), and roots collected at full maturity (FM) stages of WT and transgenic 392-3 soybean plants supplied with 10, 32, or 100 μM of Fe(III)-EDDHA.** Values are the averages of at least five samples ± SE. Asterisks indicate that the means (between WT and 392-3 within tissue type) are different by the Tukey HSD test (*P < 0.05; **P < 0.01; ***P < 0.001).

To test if the constitutive expression of *AtFRO2* and a combination of different Fe supplies would influence dry mass accumulation in soybean organs, we cultivated WT and 392-3 plants with 10, 32, or 100 μM Fe(III)-EDDHA until FM. The lowest Fe supplies (10 and 32 μM) resulted in significant differences in root dry weight (DW) between the WT and 392-3 line, while no differences were seen between genotypes in the roots at the highest Fe supply of 100 μM Fe (III)-EDDHA (**Figure 2**). The transgenic plants also had significantly heavier shoot DW (*P* < 0.001) than the WT, regardless of the Fe concentration in the growth solution. At 10 and 32 μM Fe (III)-EDDHA the transgenic soybean line 392-3 had significantly higher pod wall DW. High Fe supply caused a decrease of about 30% in root DW in the transgenic line compared to the lower concentrations (10 or 32 μM). In the WT plants, this reduction in root DW was not observed. In fact, WT plants exhibited higher root DW (12.5 ± 0.8 g and 12.2 ± 0.1g) when grown in higher Fe concentrations than when grown at 10 μM concentration (9.4 ± 0.3g).

### *AtFRO2* AND *GmFer* EXPRESSION

**FIGURE 3:**
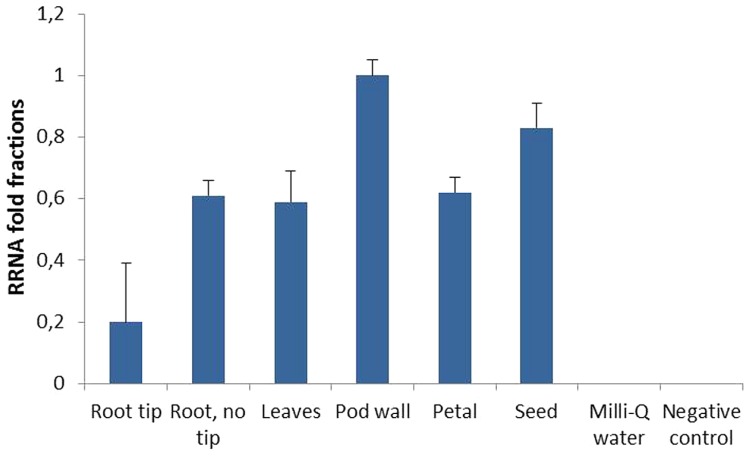
**Relative *AtFRO2* RNA expression in different soybean plant tissues measured by Quantitative RT-PCR (Biorad).** Amount of transcript was calculated according to internal 18S RNA expression for each tissue. RNA was extracted from two plants for each tissue and Quantitative RT-PCR reaction was repeated three times.

Quantitative RT-PCR revealed that the CaMV 35S promoter drove *AtFRO2* expression in different organs of the transgenic plants such as root tips, basal regions of roots (without tips), leaves, petals, pod walls, and seeds, and that *AtFRO2* transcript levels were relatively similar amongst the different organs (**Figure 3**). However, when looking at different root regions, it was found that *AtFRO2* was less expressed in root tips than in the remaining regions of the root. No expression of *AtFRO2* was found in the non-transformed control.

**FIGURE 4:**
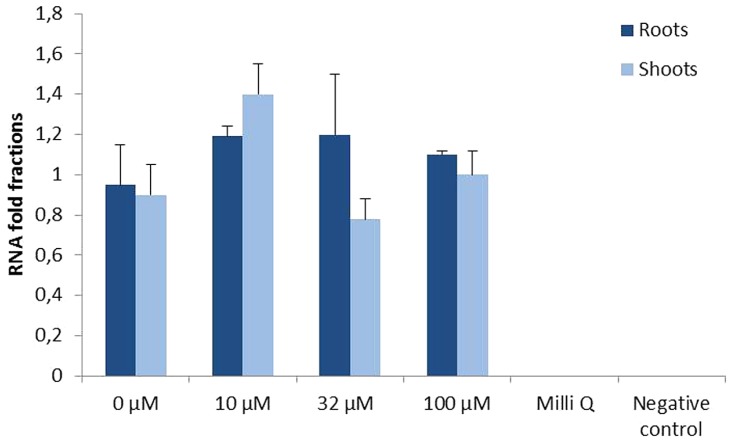
**Relative *AtFRO2* RNA expression in roots and shoots of transgenic 392-3 soybean plants supplied with 0, 10, 32, or 100 μM Fe(III) EDDHA detected by Quantitative RT-PCR (Biorad).** Amount of transcript was calculated according to internal 18S RNA expression for each tissue. RNA was extracted from 2 plants for each tissue and Quantitative RT-PCR reaction was repeated three times.

In order to determine if the constitutive expression of *AtFRO2* is influenced by the external supply of iron, quantitative RT-PCR was performed in roots and shoots of transgenic soybean line 392-3 grown at 0, 10, 32, or 100 μM Fe(III)-EDDHA for 14 days. Similar transcript levels were observed in both roots and shoots, regardless of iron concentration in the hydroponics solution (**Figure 4**).

**FIGURE 5:**
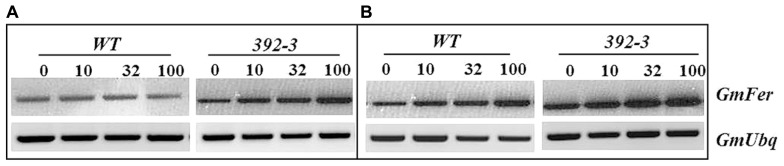
**Semi-quantitative RT-PCR analysis of the soybean ferritin (*GmFer*) expression in roots (Panel A) and shoots (Panel B) of WT and 392-3 *G.* max plants grown under different iron concentrations [0, 10, 32, or 100 μM Fe (III)-EDDHA].** Soybean ubiquitin gene (*GmUbq*) was used as template loading control.

*Ferritin* expression was assessed in roots and shoots of control and transgenic plants (**Figure 5**). Higher *ferritin* levels were found in the shoots than in the roots in both control and transgenic plants, and an increase in *ferritin* expression was found when plants were grown at higher iron concentrations. Moreover, 392-3 plants appeared to have higher expression of the *ferritin* gene when compared to control.

### TISSUE MINERAL CONCENTRATIONS

At an initial stage of our experiments, 392-3 soybeans were grown along with the WT plants for 14 days at 32 μM Fe(III)-EDDHA in hydroponic conditions and plants were screened for possible modulatory effects on mineral concentrations in roots, stems, and leaves (**Table 1**). It was observed that the transgenic line had significantly higher concentrations of Fe, Zn, P, K, and Mn in leaves, roots and stems, with concentrations increasing more than 50% when compared to the WT.

**Table 1:** Average root, stem, and leaf mineral concentration of wild-type (WT) and 392-3 transgenic soybean plants grown in hydroponic conditions with 32 μM Fe(III)-EDDHA for 2 weeks.

Mineral (μg g^-1^)	Roots		Stems		Leaves	
	WT	392-3	WT	392-3	WT	392-3
Fe	201 ± 11	582 ± 25**	41 ± 3	94 ± 6**	196 ± 8	766 ± 31**
Mn	122 ± 10	258 ± 13**	122 ± 10	258 ± 13**	65 ± 2	188 ± 8**
K	46430 ± 7979	51266 ± 2875*	33692 ± 2556	46004 ± 2467*	27244 ± 389	30575 ± 1306*
P	12284 ± 738	17490 ± 880*	4558 ± 195	6106 ± 393*	4658 ± 34	6912 ± 105*
Zn	100 ± 4	346 ± 11**	56 ± 3	149 ± 9**	136 ± 3	283 ± 6**

Values are shown as mean ± SE and represent an average of five plants. Significance within tissue types: *P < 0.05; **P < 0.01.

**FIGURE 6:**
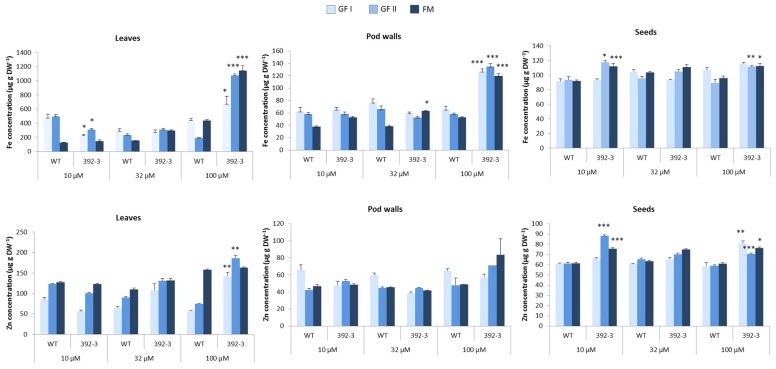
**Fe and Zn concentration (μg g^-1^) in leaves, pod walls and seeds of WT and transgenic 392-3 soybean plants grown in hydroponic conditions with 10, 32, or 100 μM Fe (III)-EDDHA.** Tissues were harvested at two grain filling stages (GF I and GF II) and at full maturity (FM). Asterisks indicate that the means (between WT and 392-3 within Fe supply) are different by the Tukey HSD test (*P < 0.05; **P < 0.01; ***P < 0.001). Error bars may be too small to be visible in the figure. Data are means ± SE of five plants per treatment.

The 392-3 soybeans were also grown along with the WT plants at 10, 32, or 100 μM Fe (III)-EDDHA in hydroponic conditions and plants were screened up to FM. Iron and Zn determinations of the leaves, pod walls and seeds were performed at three different developmental seed filling stages: Grain-Fill I (GF I), Grain-Fill II (GF II), and FM (**Figure 6**). Significant differences in Fe accumulation in the different tissues was found when comparing WT and 392-3 plants, and differences were particularly significant at the last collection date (FM), and at the highest iron concentration (100 μM). For plants that were grown at 100 μM Fe, at the last collection date (**Table 2**; **Figure 6**) 392-3 plants had up to 1142 ± 69 μg g^-1^ Fe in the leaves, whereas the WT plants had 435 ± 18 μg g^-1^. Nonetheless, the increase in Fe concentration in the leaves of plants grown at 100 μM Fe (III)-EDDHA was already significant at the GF I and GF II stages. The pod wall Fe levels also were significantly higher in the 392-3 plants, relative to WT, at 32 and 100 μM Fe growth conditions at FM (**Figure 6**), with values reaching up to 120 ± 4 μg g^-1^. At FM, an increase of up to 100% was found in iron levels in the leaves of plants grown at 100 μM Fe(III)-EDDHA, a higher than 60% increase was found in the levels of iron in the pod walls of these same plants, and a 10% increase was detected in the corresponding seed iron levels. Plants that had been grown in soil, in greenhouse conditions, showed a similar increase in seed iron levels (data not shown).

**Table 2:** Mineral concentration (μg g^-1^) in roots, pod walls, leaves, and seeds of wild-type (WT) and transgenic 392-3 soybean plants grown in hydroponic conditions with 100 μM Fe(III)-EDDHA until full maturity (FM).

Mineral (μg g^-1^)	Roots		Pod walls		Leaves		Seeds	
	WT	392-3	WT	392-3	WT	392-3	WT	392-3
Fe	2904 ± 109	2592 ± 177	49 ± 1	120 ± 4***	435 ± 18	1142 ± 69***	96 ± 2	112 ± 3**
Mn	10 ± 0.6	9 ± 0.3	22 ± 2	17 ± 1	49 ± 6	47 ± 1	20 ± 1	24 ± 1*
K	13256 ± 83	41661 ± 2644*	26722 ± 436	32064 ± 620**	15385 ± 200	13336 ± 977	18920 ± 415	19341 ± 78
P	4079 ± 97	7991 ± 529*	2572 ± 37	4244 ± 89**	1427 ± 53	2414 ± 68**	6389 ± 164	6509 ± 132
Zn	45 ± 1	137 ± 50*	49 ± 1	84 ± 19**	157 ± 3	162 ± 2	61 ± 1	76 ± 1**
Cu	27 ± 1	25 ± 2	8 ± 0.1	13 ± 0.3**	3 ± 0.1	8 ± 0.4**	15 ± 0.1	17 ± 0.1***
Ca	9788 ± 72	7553 ± 200*	23605 ± 395	19275 ± 124**	21861 ± 330	30398 ± 591**	3519 ± 57	3674 ± 61
Ni	2 ± 0.4	3 ± 0.2*	3 ± 0.1	5 ± 0.1**	1 ± 0.1	2 ± 0.2**	8 ± 0.1	9 ± 0.1*
Mg	435 ± 0.5	534 ± 12*	4886 ± 61	4310 ± 59*	1648 ± 28	2346 ± 120*	2190 ± 21	2103 ± 16
Mo	5 ± 0.3	3 ± 0.3*	39 ± 2	20 ± 1	23 ± 2	18 ± 1	55 ± 0.9	55 ± 0.3

Values are shown as mean ± SE and represent an average of five plants. Significance within tissue types: *P < 0.05; **P < 0.01; ***P < 0.001.

Zinc concentrations were also higher in the transgenic leaves and seeds but not in the pod walls. The increments were varied with the concentration of iron in the nutrient solution and with the harvest date. In the leaves, increases in Zn concentration were significantly higher at GF I and GF II, but not at FM. In the seeds, Zn concentration in 392-3 was always significantly higher than in WT plants grown at the highest Fe concentration.

Because Fe and Zn differences were more pronounced in plants grown at 100 μM Fe and at FM, we decided to determine whether other minerals were also affected, looking at this particular stage of development and treatment. **Table 2** shows that other minerals besides Fe and Zn were also modulated in the transgenic plants, with differences noted in mineral composition in the roots, pod walls, leaves, and seeds (**Table 2**). In roots, several obvious mineral concentration differences could be detected. High Fe supply, in combination with the constitutive expression of *AtFRO2*, was associated with higher concentrations of K, P, Zn, Ca, Ni, Mg, and Mo. In pod walls, transgenic plants had significantly higher Fe, K, P, Cu, and Ni concentrations and significantly lower concentrations of Ca and Mg. In leaves there was a significant increase in leaf Fe, but also in P, Cu, Ca, Ni, and Mg. In seeds, the concentrations of Fe, Zn, Cu, and Ni were significantly increased in the transgenic line, relative to WT.

### MINERAL CORRELATION ANALYSIS

**FIGURE 7:**
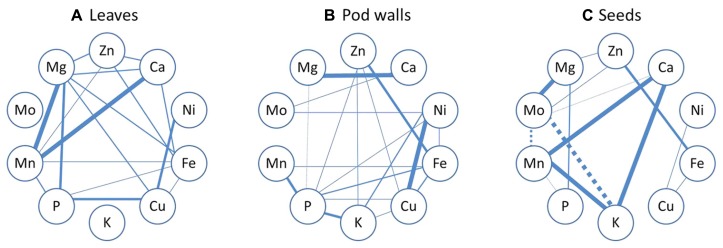
**Mineral correlation analysis.** Pearson’s correlation analysis of ten mineral concentrations in leaves **(A)**, pod walls **(B)**, and seeds **(C)** of Thorne and 392-3 soybean plants cultivated with 10, 32, or 100 μM of Fe (III)-EDDHA and measured at two grain filling stages (GF I and GF II) or at full maturity (FM). Solid lines represent a significant positive correlation and dashed lines represent a significant negative correlation. Thinner lines indicate significance at the *P* < 0.05, semi-thin indicate significance at the *P* < 0.01, semi-thick indicate significance at the *P* < 0.001 level and the thicker lines indicate significance at the *P* < 0.0001 level.

Pearson’s correlation analysis was performed in order to find relationships among the ten minerals’ concentrations in three different plant tissues. The organ with the fewest number of correlations was the seed; however, it was also the organ where the most significant correlations occurred (**Figure 7**). In general, Fe seems to be very tightly linked to several other minerals, with particular emphasis in the leaves and pod walls. As can be observed in **Figure 7**, the only positive correlations that are common for the three tissue types are the pairs Fe-Zn, Fe-Cu, and Ni-Cu. Other correlations are common in two of the three tissues.

### LEAF IRON REDUCTION ACTIVITY

**FIGURE 8:**
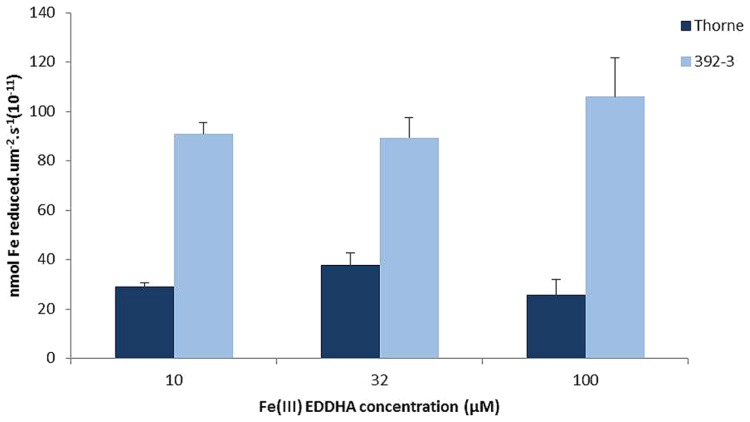
**Iron reductase activity measured *in vivo* in protoplasts isolated from WT and transgenic 392-3 soybean leaves of plants grown with 10, 32, or 100 μM Fe (III)-EDDHA.** Values are shown per surface area. Measurements were made with 400 μM Fe (III)-EDTA as a substrate. Data are means ± SE of three replications each.

The small differences in the seed iron concentration between WT and 392-3 transgenic plants led to the hypothesis that perhaps the higher reductase activity in the roots may not be paralleled by a higher reductase activity in the leaves or in other plant organs, which could be sources of transportable Fe. If the increased iron concentration in the leaves of the 392-3 plants is being stored in a non-transportable form, then there will not be a concomitant transport to the developing seeds. Therefore, an optimized protocol was used to measure ferric chelate reductase activity of soybean plants grown in hydroponics for two weeks at 10, 32, or 100 μM Fe (III)-EDDHA. Protoplasts isolated from leaf cells of 392-3 and WT plants showed that expression of *AtFRO2* increased leaf iron reduction capacity up to 3-fold when compared to the WT, regardless of the plant iron treatment (**Figure 8**).

### CHANGES IN LEAF AND ROOT ORGANIC ACIDS

**FIGURE 9:**
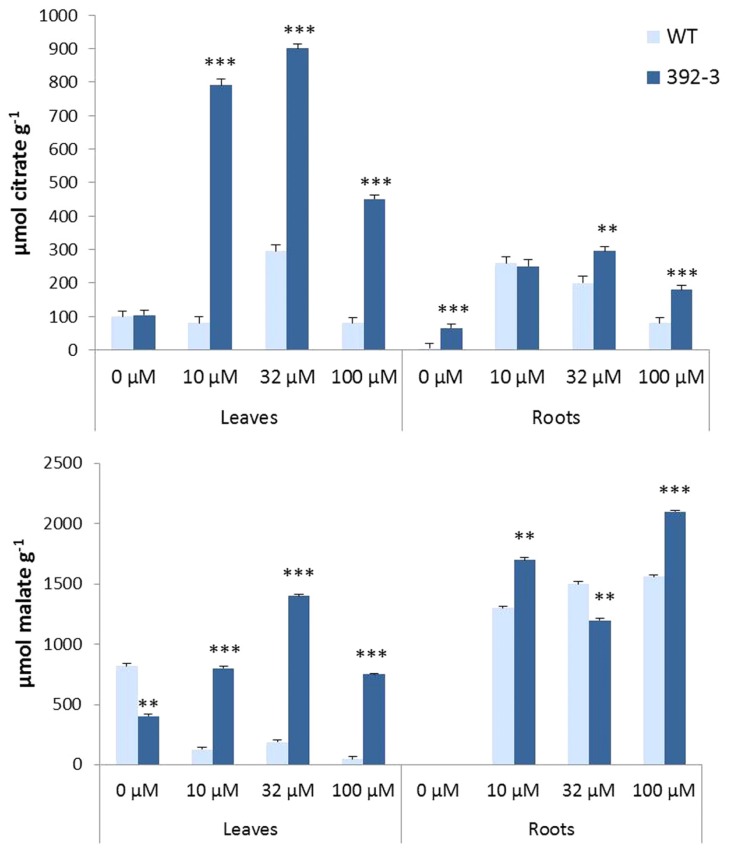
**Effects of Fe concentration on organic acid (citrate and malate) accumulation in WT and 392-3 soybean leaves and roots.** Data are means ± SE of eight replicates. Asterisks represent significant differences between WT and 392-3 (**P < 0.01; ***P < 0.001).

Malate and citrate have been described as important compounds in the translocation of Fe in the plant’s vascular system. Both OA were detected in transgenic and control leaves and roots. In general, differences between 392-3 and WT plants were more pronounced in the leaves than in the roots, and transgenic plants accumulated significantly higher amounts of both OA than WT. In the leaves of 392-3 plants, citrate and malate were higher when plants were grown at 10, 32, or 100 μM Fe (**Figures 9A,B**). A different pattern was observed in the WT plants: malate concentrations were highest in leaves of iron-starved plants, and were lower when iron was available to the plants. Leaf citrate concentrations were only elevated at 32 μM Fe.

For plants grown in the absence of Fe, citrate, and malate were not detectable in the roots of WT and transgenic plants. However, when plants were grown in Fe sufficiency, both OA were observed (**Figures 9A,B**).

## DISCUSSION

There is currently a strong interest in developing strategies to increase the level of minerals, such as iron, in edible plant organs. One such strategy is the over-expression of genes that are necessary for proper plant iron status. Previous work has shown that by enhancing the reductase activity in transgenic soybean, plants are able to cope better in iron limiting soils, having higher chlorophyll values and improved agronomic performance (Vasconcelos et al., 2006). Also, a possible strategy to enhance the mineral content of plant foods is to enhance the uptake of minerals from the roots during the period of seed development (Waters and Grusak, 2008; Sperotto et al., 2012).

### MINERAL CONCENTRATIONS AND CORRELATIONS

In the current study, soybean plants overexpressing *AtFRO2* were analyzed for mineral concentrations (Fe, Zn, Cu, Mn, Mg, Ca, Mo, Ni, K, and P) in source and sink tissues at different seed-filling stages. Significant differences were found in the mineral concentration of transgenic plants when compared to the WT, and differences were particularly prominent when tissues were collected at the final developmental stage (**Table 1**). This suggests that the iron reductase is not only involved in iron acquisition, but it plays a more general role in the regulation of ion absorption by root cells. This hypothesis has been supported by others (Marschner et al., 1982; Stephan and Grün, 1989; Welch and LaRue, 1990; Yi and Guerinot, 1996). Furthermore, after two weeks in hydroponic conditions supplemented with 0, 10, 32, or 100 μM Fe (III)-EDDHA, the transgenic line 392-3 had significantly higher concentrations of Fe, Zn, and Mn in leaves, roots, and stems, with concentrations 50% higher when compared to the WT (**Table 1**). Increased Mn uptake and subsequent translocation to the aerial parts of the plant is reasonable, because Mn moves easily from roots to shoot tissues in the xylem-sap transpiration stream (Ramani and Kannan, 1987; Marschner, 1995).

After observing that 392-3 had altered concentrations of different minerals after 14 days of growth, we grew plants to maturity and analyzed mineral accumulation in roots, leaves, pod walls, and seeds at different developmental seed filling stages (**Figure 6**). At the last harvest date (FM), it was observed that when grown with high Fe supply, in combination with the constitutive expression of *AtFRO2*, there were several significant mineral changes. In pod walls, transgenic plants had significantly higher Fe, K, P, Cu, and Ni; however, significantly lower concentrations of Ca and Mg were found (**Table 2**), suggesting an antagonistic effect of the higher Fe concentrations in these tissues, or that pod transpiration rates were depressed. Finally, besides Fe, seeds had also significantly higher concentrations of Zn, Cu, and Ni.

There were two minerals which were significantly higher across all tissues of 392-3 when compared to WT (roots, leaves, pod walls, and seeds): Ni and P. In fact, Ni rapidly re-translocates from leaves to young tissues in the phloem, particularly during reproductive growth. Up to 70% of Ni in the shoots is transported to the seeds of soybean (Tiffin, 1971).

In the case of P, more P becomes available for uptake when there is an increase of OA exudation (Remy et al., 2012). Thus, our observations of significantly higher OA production by 392-3 roots and leaves (**Figure 9**) could explain the increased uptake of P. If this hypothesis is true, then it suggests that the most important source of P for the seeds is the P taken up during seed fill, and not remobilized P. In fact, in previous work where P was either remobilized or not from the leaves, no changes in P occurred in soybean seeds (Crafts-Brandner, 1992).

When looking at mineral correlations (**Figure 7**), the positive correlations which were common in leaves, pod walls and seeds were the pairs Fe-Zn, Fe-Cu, and Ni-Cu. Ni-Cu share a common uptake system (Kochian, 1991), and Fe-Zn as well, because the IRT1 Fe transporter also mediates Zn transport (Eide et al., 1996; Korshunova et al., 1999; Vert et al., 2002). The link between Fe-Cu cannot be explained by common transporters, as it has been shown that IRT1 (at least in *Arabidopsis*) cannot transport Cu. However, *35S-FRO2* transgenic *Arabidopsis* plants have elevated Cu reductase activity (Connolly et al., 2003; Zimmermann et al., 2009), and at 14d 392-3 plants also have enhanced Cu reductase activity (data not shown), which may have increased the amount of available Cu for uptake, transport and accumulation.

In leaves and pod walls, there was a positive correlation between Fe-Mn, contrary to what was found in rice (Sperotto et al., 2012). In seeds, there was a positive correlation between Ca and Mn, which is in agreement with Zeng et al. (2005) and Majumder et al. (1990). Also in the seeds, there was a positive correlation between Ca and K, confirming what was seen before in rice (Sperotto et al., 2012).

The mineral correlations in the different plant organs could also be related to differential mobilities within the plant, and several mineral elements, such as Ca and Mo, are not very phloem mobile (Marschner, 1995; Lucas et al., 2013). Once deposited in the leaves, minerals must be remobilized to the seeds via phloem transport, and K, Na, Mg, P, are transported readily, but Fe, Zn, Cu, and Mo are less mobile, and Mn and Ca are poorly mobile in the phloem of most plant species (Marschner, 1995). Mineral elements that have low phloem mobilities generally accumulate in tissues with high transpiration rates (White and Broadley, 2009).

### THE ROLE OF SHOOT Fe REDUCTASE ACTIVITY

An interesting question that arises from this study is why 392-3 acquired only 10% more iron in the seeds when leaves and pod walls were more highly enriched in iron. Why did this excess iron not move to the seeds? It has been shown previously that in soybean (Lazlo, 1990), the developing ovules exhibit a gradual increase in seed Fe accretion as long as seed DW is increasing. In the current study, the source tissues of the transgenic plants showed more than double the iron concentration when compared to control plants (**Table 1**), and in certain instances, a 5-fold increase was observed in iron concentrations in the leaves. The small (yet significant) differences in the seed iron concentration between WT and 392-3 plants suggested that perhaps the higher reductase activity in the roots (Vasconcelos et al., 2006) may not have been paralleled by a higher reductase activity in the leaves. However, protoplasts isolated from leaf cells of 392-3 and WT plants showed that *AtFRO2* expression increased leaf iron reduction capacity up to 3-fold, relative to WT (**Figure 1**). However, it appears that having higher iron reduction capacity in the leaves (and presumably other vegetative tissues) may confer only a modest benefit in the availability and/or loading of iron to the phloem and subsequent transport to the seeds. Fe remobilization from leaves to seeds has been seen in legumes (Hocking and Pate, 1977; Grusak, 1994), and remobilization from leaves to seeds increases during senescence in common bean (*Phaseolus vulgaris* L.; Zhang et al., 1995). At FM, in the current study, we already had several senescing leaves, which should be working as sources of Fe to the seeds.

Chelation is a required factor for phloem transport of iron. Using the *brz* and *dgl* Fe-hyperaccumulating pea mutants, it was suggested that Fe must be chelated prior to phloem loading, since transition metal ions precipitate at alkaline pH values characteristic of phloem saps (Grusak, 1994). It is possible that our transgenic 392-3 soybean plants did not produce enough chelators to transport the excess leaf iron to the seeds. Once iron has entered the plant, both NA and citrate have been proposed to serve as iron chelators; mutants that do not properly synthesize or transport these chelators have lower Fe accumulation in the seeds (Jeong et al., 2008). We have shown that the 392-3 plants have significantly higher citrate and malate concentrations in leaves and roots. Several authors have reported an increase in xylem sap OA concentrations with Fe deficiency (Abadía et al., 2002; López-Millán et al., 2009; Larbi et al., 2010), a condition where there is up-regulation of the Fe reductase. Also, FRO2 belongs to a superfamily of flavocytochrome oxidoreductases, containing a NADPH sequence motif on the inside of the membrane (Schagerlof et al., 2006). It is possible that elevated levels of AtFRO2 lead to increased NADPH consumption, which consequently induces metabolic pathways that lead to a higher production of OA (López-Millán et al., 2000). Because the higher accumulation of malate and citrate did not enable increased seed iron levels, it seems that citrate and malate may not be limiting factors contributing to the transport of Fe to seeds.

Perhaps other factors need to be turned on for higher phloem Fe translocation from shoots to seeds. In rice, it is known that OsYSL2 expression is a necessary component for correct translocation of Fe to young shoots and developing seeds (Ishimaru et al., 2010; Masuda et al., 2012), as it is thought to transport Fe(II)-NA in the phloem (Koike et al., 2004). If there is a similar need in soybean, it is possible that the expression of this ortholog in our transgenic plants may not have been up-regulated.

### IRON STORED AS FERRITIN

Another factor that could have prevented the remobilization of Fe to the seeds is if Fe was stored in a non-translocatable form, e.g., complexed within ferritin. Ferritin is one of the principal forms of iron storage in plants, and it provides a means of rapidly sequestering iron ions that might otherwise promote the formation of reactive oxygen species (Truty et al., 2001). *Ferritin* expression was assessed in roots and shoots of control and transgenic plants in order to see if the extra iron could be stored in the form of ferritin (**Figure 3**). Higher *ferritin* transcript levels were found in the shoots than in the roots in both control and transgenic plants, and an increase in *ferritin* expression was found when plants were grown at higher iron concentrations (100 μM Fe). Moreover, 392-3 plants seemed to have higher expression of the *ferritin* gene, indicating that the excess iron concentration found in the roots and shoots of the transgenic plants is at least partly stored in the form of ferritin, and thus may not be readily available for export.

## CONCLUSION

Accumulation of iron in the various plant tissues during growth and development is a dynamic process resulting from an integrated regulation of genes encoding proteins for mineral uptake, transport and storage. These processes depend on the plant genotype and are greatly influenced by environmental cues. Can an improved soybean be developed that is fortified with essential minerals? The results presented herein demonstrate that constitutive expression of an iron reductase gene led to a 10% increase in seed Fe, Zn, and Cu, and a 20% increase in Mn levels, despite the fact that mineral concentration in the leaves and pod walls (two of the most important mineral sources for the seeds) reached much higher relative levels in the transgenic plants. This indicates that manipulation of the iron reductase could be an effective biofortification strategy for several minerals, especially when targeting leafy food sources.

## Conflict of Interest Statement

The authors declare that the research was conducted in the absence of any commercial or financial relationships that could be construed as a potential conflict of interest.
